# Food Insecurity and Risk of Dementia and Cognitive Impairment With No Dementia in US Older Adults

**DOI:** 10.1001/jamanetworkopen.2025.33592

**Published:** 2025-09-24

**Authors:** Heejin Lee, Elizabeth Ludwig-Borycz, Steven G. Heeringa, Lindsay H. Ryan, Kenneth M. Langa, Claire T. McEvoy, Julia A. Wolfson, Cindy W. Leung

**Affiliations:** 1 Department of Nutrition, Harvard T.H. Chan School of Public Health, Boston, Massachusetts; 2 Center for Global Health Equity, University of Michigan, Ann Arbor; 3 Institute for Social Research, University of Michigan, Ann Arbor; 4 Department of Internal Medicine, University of Michigan Medical School, Ann Arbor; 5 Centre for Public Health, Institute for Global Food Security, Queen’s University Belfast, Northern Ireland, United Kingdom; 6 Department of Health Policy and Management, Johns Hopkins Bloomberg School of Public Health, Baltimore, Maryland; 7 Department of International Health, Johns Hopkins Bloomberg School of Public Health, Baltimore, Maryland

## Abstract

This cohort study examines the associations between food insecurity and the risks of dementia, cognitive impairment with no dementia, and cognitive impairment with dementia among US older adults.

## Introduction

The prevalence of Alzheimer disease and related dementias among US older adults is expected to increase by 2060.^1^ Food insecurity may lead to impaired cognitive function and increased dementia risk.^2,3^ Few studies have examined longitudinal associations between food insecurity and serveral cognitive outcomes. Using nationally representative longitudinal data, we examined the associations between food insecurity and the risks of dementia, cognitive impairment with no dementia (CIND), and cognitive impairment with dementia.

## Methods

Data were obtained from the Health and Retirement Study (HRS). The University of Michigan institutional review board approved the protocol, and all participants provided informed consent. Participants (5851 with dementia, 4886 with CIND, 5129 with cognitive impairment with dementia) aged 50 years or older, without dementia or memory problems at baseline and without developing any condition of interest before the 2014 HRS, were followed up until the 2020 HRS. Analyses of CIND excluded incident dementia during follow-up (eFigure in Supplement 1). This study followed the STROBE reporting guideline for cohort studies.

Food insecurity was assessed in the 2013 HRS Health Care and Nutrition Study and categorized using the validated 6-Item Food Security Survey Module,^4^ as high (0-1), low (2-4), and very low (5-6) food security. Cognitive outcomes were defined based on the validated Langa-Weir classification,^5^ using biennial cognitive assessments for dementia (scores ≤6), CIND (scores 7-11), and cognitive impairment with dementia (scores ≤11). Person-years were calculated from entry to first identification of cognitive impairment, death, loss to follow-up, or end of follow-up.

Multivariable-adjusted Cox proportional hazards regression models, incorporating attrition-adjusted sampling weights, examined associations between food insecurity and risk of cognitive impairment. Subgroup analyses were stratified by age. Hazard ratios (HRs) with 95% CIs were estimated. A 2-sided *P* < .05 was considered statistically significant. Data were analyzed using SAS, version 9.4 (SAS Institute Inc) (eMethods in Supplement 1).

## Results

In the main analytic sample (n = 5851; mean [SD] age, 67.1 [9.8] years; 3472 women and 2379 men), 546 of 5851 (9.3%) had low food security, and 411 of 5851 (7.0%) had very low food security. During follow-up (median, 8.7 years [IQR, 6.3-8.9 years]), 423 dementia cases, 972 CIND cases, and 1215 cases of cognitive impairment with dementia occurred.

Compared with food security, low food security (HR, 1.72; 95% CI, 1.20-2.46) was associated with higher dementia risk (*P* = .04 for trend) (Table 1). Low food security (HR, 1.68; 95% CI, 1.24-2.31) and very low food security (HR, 1.79; 95% CI, 1.25-2.57) were associated with CIND (*P* < .001 for trend). Low food security (HR, 1.61; 95% CI, 1.24-2.10) and very low food security (HR, 1.65; 95% CI, 1.18-2.31) were associated with cognitive impairment with dementia (*P* = .001 for trend). Associations between food insecurity and cognitive outcomes were more prominent among participants younger than 65 years at baseline (Table 2).

**Table 1.** Estimated Multivariable-Adjusted Hazard Ratios for Dementia, Cognitive Impairment With No Dementia, and Cognitive Impairment With Dementia Risk According to Food Insecurity Status^a^

Cognitive outcome	Adjusted hazard ratio (95% CI)			*P* value for trend
	Food secure	Low food secure	Very low food secure	
**Dementia**				
No. of cases/person-years	327/36 987	58/4090	38/3050	NA
Multivariable adjusted 1^b^	1.00 [Reference]	1.86 (1.32-2.60)	1.61 (1.08-2.40)	.002
Multivariable adjusted 2^c^	1.00 [Reference]	1.78 (1.25-2.51)	1.42 (0.95-2.13)	.01
Multivariable adjusted 3^d^	1.00 [Reference]	1.72 (1.20-2.46)	1.31 (0.88-1.93)	.04
**Cognitive impairment with no dementia**				
No. of cases/person-years	750/30 299	129/2814	93/2179	NA
Multivariable-adjusted 1^b^	1.00 [Reference]	1.76 (1.33-2.35)	2.03 (1.43-2.89)	<.001
Multivariable-adjusted 2^c^	1.00 [Reference]	1.72 (1.28-2.32)	1.95 (1.37-2.79)	<.001
Multivariable-adjusted 3^d^	1.00 [Reference]	1.68 (1.24-2.31)	1.79 (1.25-2.57)	<.001
**Cognitive impairment with dementia**				
No. of cases/person-years	944/31 159	159/2941	112/2242	NA
Multivariable-adjusted 1^b^	1.00 [Reference]	1.69 (1.33-2.15)	1.87 (1.34-2.60)	<.001
Multivariable-adjusted 2^c^	1.00 [Reference]	1.67 (1.29-2.15)	1.78 (1.27-2.50)	<.001
Multivariable-adjusted 3^d^	1.00 [Reference]	1.61 (1.24-2.10)	1.65 (1.18-2.31)	.001

Abbreviation: NA, not applicable.

^a^ Food insecurity was categorized as follows: a score of 0 to 1 indicates food secure; 2 to 4, low food secure; and 5 to 6, very low food secure.

^b^ Models were adjusted for age (continuous, years), sex (male, female), and race and ethnicity (Black, Hispanic, White, and other [Alaskan Native, American Indian, Asian, and Pacific Islander]), marital status (never married, married but spouse absent, separated, divorced, widowed, married or living with a partner), current employment status (full time, part time, partly retired; retired; unemployed, disabled, and not in the labor force), educational level (less than high school, high school graduate, some college or college graduate, postcollege), total net worth (tertile), and household size (1, 2, or ≥3 members). Given the higher prevalence of food insecurity among Black and Hispanic individuals, we included race and ethnicity in the model.

^c^ Models were further adjusted for energy intake (continuous, kilocalories per day), vigorous activity (yes or no), smoking (never smoker, ever smoker, current smoker), alcohol consumption (nondrinker, <5 g/d, ≥5 g/d), and body mass index (<25, 25-30, ≥30 [calculated as weight in kilograms divided by height in meters squared]).

^d^ Models were further adjusted for disease history (yes or no; disease included diabetes, heart problems, stroke, cancer, and lung disease) and baseline depressive symptoms (yes or no; 8-item Center for Epidemiologic Studies Depression Scale score ≥5). All models accounted for the complex sampling design of the Health and Retirement Study, incorporating attrition-adjusted sampling weights.

**Table 2.** Estimated Multivariable-Adjusted Associations Between Food Insecurity and Incidence of Dementia, Cognitive Impairment With No Dementia, and Cognitive Impairment With Dementia Stratified by Baseline Age^a^^,^^b^

Cognitive outcome	No. of cases/person-years	Adjusted hazard ratio (95% CI)			*P* value	
		Food secure	Low food secure	Very low food secure	Trend	Interaction
**Dementia**						
Age <65 y	84/18 576	1.00 [Reference]	2.80 (1.20-6.51)	1.57 (0.73-3.44)	.02	.005
Age ≥65 y	339/23 151	1.00 [Reference]	1.26 (0.81-1.95)	1.05 (0.58-1.92)	.34	
**Cognitive impairment with no dementia**						
Age <65 y	355/17 955	1.00 [Reference]	2.20 (1.38-3.49)	1.89 (1.20-2.97)	.003	.01
Age ≥65 y	617/17 338	1.00 [Reference]	1.09 (0.74-1.61)	1.85 (1.13-3.02)	.65	
**Cognitive impairment with dementia**						
Age <65 y	401/18 161	1.00 [Reference]	2.16 (1.40-3.33)	1.75 (1.12-2.72)	.001	.001
Age ≥65 y	814/18 181	1.00 [Reference]	1.12 (0.81-1.55)	1.54 (0.99-2.39)	.50	

^a^ Food insecurity was categorized as follows: a score of 0 to 1 indicates food secure; 2 to 4, low food secure; and 5 to 6, very low food secure.

^b^ Models were adjusted for age (continuous, years), race and ethnicity (Black, Hispanic, White, and other [Alaskan Native, American Indian, Asian, and Pacific Islander]), sex (male, female), marital status (never married, married but spouse absent, separated, divorced, widowed, married or living with a partner), current employment status (full time, part time, partly retired; retired; unemployed, disabled, and not in the labor force), educational level (less than high school, high school graduate, some college or college graduate, postcollege), total net worth (tertile), household size (1, 2, or ≥3 members), energy intake (continuous, kilocalories per day), vigorous activity (yes or no), smoking (never smoker, ever smoker, current smoker), alcohol consumption (nondrinker, <5 g/d, ≥ 5g/d), body mass index (<25, 25-30, ≥30 [calculated as weight in kilograms divided by height in meters squared]), disease history (yes or no; disease included diabetes, heart problem, stroke, cancer, and lung disease), and baseline depressive symptom (yes or no; 8-item Center for Epidemiologic Studies Depression Scale score ≥5), and accounted for the complex sampling design of the Health and Retirement Study, incorporating attrition-adjusted sampling weights. Given the higher prevalence of food insecurity among Black and Hispanic individuals, we included race and ethnicity in the model.

## Discussion

This nationally representative longitudinal cohort study found that food insecurity was associated with higher risks of incident dementia, CIND, and cognitive impairment with dementia, with more pronounced associations among younger adults. Our results extend prior findings from HRS^2,6^ by considering dementia and CIND separately, using the validated Langa-Weir classification,^5^ extending follow-up through 2020, and examining interaction with age.

Given the long preclinical stage of dementia, separate and combined analyses of CIND and dementia may enable more comprehensive approaches for early prevention while capturing the continuum of cognitive deterioration. Stronger associations among older adults younger than 65 years suggest that this subgroup may gain greater cognitive benefits from interventions targeting food insecurity. These results reinforce the importance of nutrition assistance programs for older adults, including congregate meal programs and the Supplemental Nutrition Assistance Program.

This study is limited by unmeasured or residual confounding and by food insecurity being assessed once with a validated measure. Future studies that can better capture long-term food insecurity and differentiate between acute and long-term food insecurity are warranted. Our findings suggest that policies addressing food insecurity may reduce the risk of cognitive impairment among aging adults.

## Notes

Supplement 1. **eFigure.** Flowchart of the Health and Retirement Study (HRS) Participants Included in the Analysis
eMethods.

eReferences.

Supplement 2.
Data Sharing Statement
