# Supplementary material for: Food Insecurity and Risk of Dementia and Cognitive Impairment With No Dementia in US Older Adults
Source: JAMA Netw Open. 2025 Sep 24;8(9):e2533592. doi: 10.1001/jamanetworkopen.2025.33592 (PMC12461400; doi:10.1001/jamanetworkopen.2025.33592)
Supplement: Supplement 1. — eFigure. Flowchart of the Health and Retirement Study (HRS) Participants Included in the Analysis eMethods. eReferences. [file jamanetwopen-e2533592-s001.pdf]

Supplementary Online Content

Lee H, Ludwig-Borycz E, Heeringa SG, et al. Food insecurity and risk of dementia and cognitive impairment with no dementia in US older adults. *JAMA Netw Open.* 2025;8(9):e2533592. doi:10.1001/jamanetworkopen.2025.33592

eFigure. Flowchart of the Health and Retirement Study (HRS) Participants Included in the Analysis

eMethods.

eReferences.

This supplementary material has been provided by the authors to give readers additional information about their work.

© 2025 Lee H et al. *JAMA Network Open.*

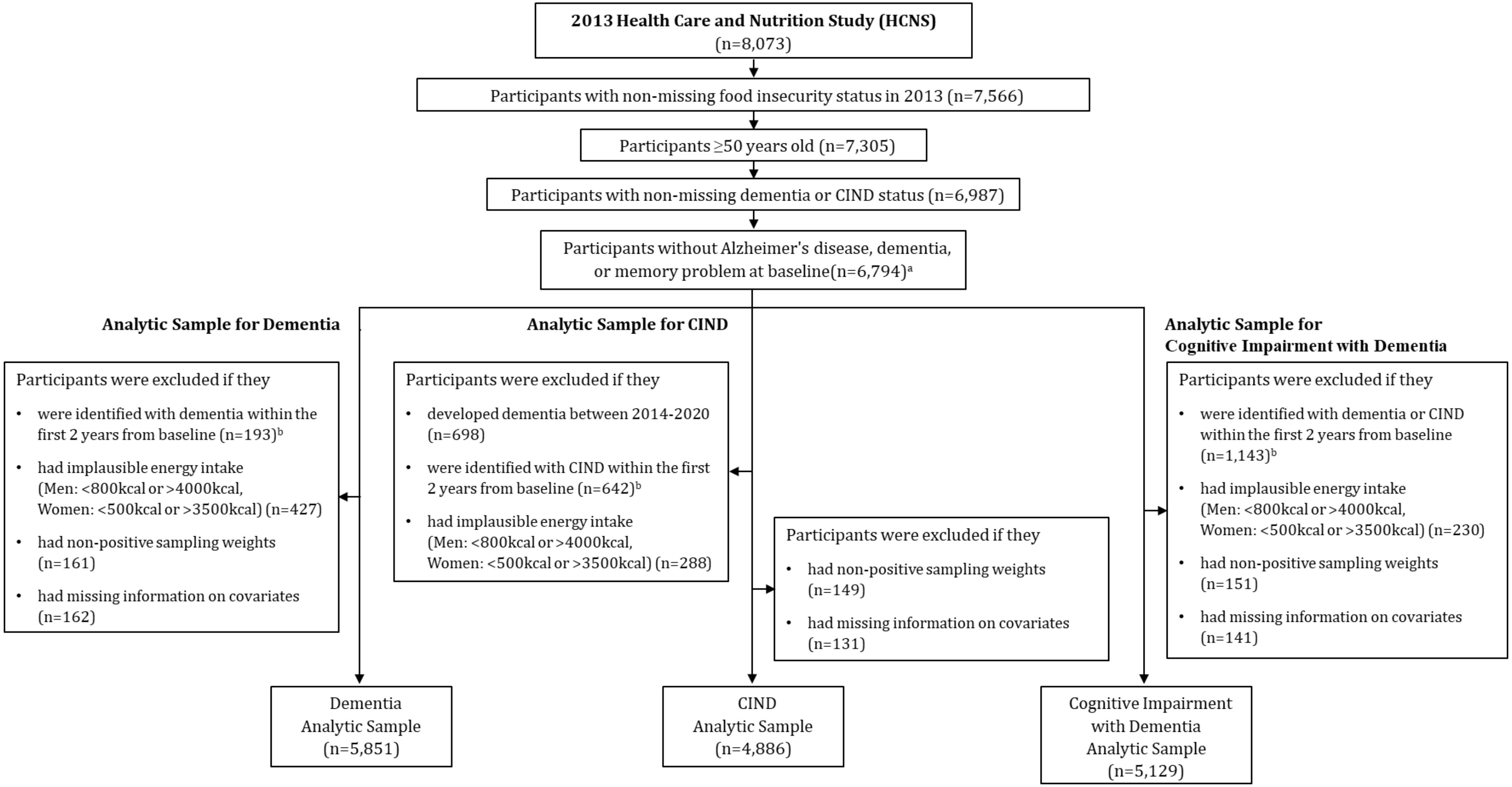

eFigure. Flowchart of the Health and Retirement Study (HRS) Participants Included in the Analysis

Abbreviations: CIND, Cognitive impairment with no dementia.

[a]Participants who reported a previous diagnosis of Alzheimer's disease, dementia, or memory problem at baseline.

[b]Participants who were identified as having dementia or CIND during the biennial HRS Core interview using the Telephone Interview for Cognitive Status and classified according to the Langa-Weir classification of cognitive function.

© 2025 Lee H et al. *JAMA Network Open.*

eMethods.

1. Study participants

The Health and Retirement Study (HRS) is a U.S. nationally representative longitudinal ongoing survey for adults aged 50 years or older and their spouse/partner. The HRS has been conducted biennially, with additional irregular off-year surveys, including 2013 HRS Health Care and Nutrition Study (HCNS). The HRS has been supported by the National Institute on Aging (NIA U01AG009740). The University of Michigan Institutional Review Board approved the protocol, and participants provided verbal or written informed consent. Participants aged 50 years or older from the 2012-2020 HRS had no dementia or memory problems at baseline (2012) and did not develop with any condition of interest prior to the 2014 HRS interview. The analysis of CIND additionally excluded participants who developed dementia during follow-up. We additionally excluded participants with implausible energy intake (male: <800 kcal/day or >4,000 kcal/day, female: <500 kcal/day or >3,500 kcal/day), with non-positive sampling weights, or with missing covariate information (in the main analytic sample: marital status n=22; educational attainment n=27; vigorous activity n=2; smoking status n=29; depressive symptoms n=82). The analytic samples yielded 5,851 participants for dementia set, 4,886 participants for cognitive impairment with no dementia set, and 5,129 participants for cognitive impairment with dementia set **(eFigure 1)**. The median (IQR) follow-up duration was 8.7 years (6.3–8.9 years) for the dementia analytic sample, 8.6 years (5.7–8.9 years) for the CIND sample, and 8.6 years (5.2–8.8 years) for the cognitive impairment with dementia sample. In the main analytic sample (n = 5,851), the weighted mean (SE) age of participants was 64.9 (0.3) years. Of the sample, 55.0% were women, and the racial and ethnic distribution was as follows: 80.6% White, 8.8% Black, 7.4% Hispanic, and 3.2% Other: American Indian, Alaskan Native, Asian, and Pacific Islander, based on weighted frequencies.

2. Food insecurity

Food insecurity was assessed using the validated USDA Six-item Food Security Survey Module[1] in the 2013 HCNS. Affirmative answers are summed with a score range of 0–6 and categorized as high (0–1), low (2–4), and very low (5–6) food security, according to USDA guidelines. In this study, food insecurity was defined to include both low food security and very low food security.

3. Cognitive Impairment

Cognitive outcomes were defined based on the validated Langa-Weir classification of cognitive function[2]. Cognitive function was assessed biennially in the HRS Core interview using adapted Telephone Interview for Cognitive Status (TICS) tests including immediate and delayed word recall, serial 7s, and backward counting. Scores from individual tests were summed to generate a total score ranging from 0 to 27. Scores of ≤6 were classified as dementia, 7–11 as CIND, and 12–27 as normal; scores of ≤11 were considered cognitive impairment with dementia. Person-years were calculated from the entry date to the first identification of cognitive impairment outcome of interest (dementia, CIND, or cognitive impairment with dementia), death, loss to follow-up, or end of follow-up (May 2021), whichever came first.

4. Covariates

Demographic covariates (age, sex, and race and ethnicity) were obtained from the 2013 HCNS, while other covariates from baseline core interviews (2012 HRS). Participants were initially asked about their Hispanic or Latino identity. If affirmative, they were asked to specify whether they were Mexican American, Puerto Rican, Cuban American, or another Hispanic subgroup. Finally, they were asked to report their race as White, Black or African American, American Indian or Alaska Native, Asian, Native Hawaiian or Pacific Islander, or other. Demographic covariates were categorized and provided by HRS as follows: sex (male, female), race and ethnicity (Black, Hispanic, White, and Other [Alaskan Native, American Indian, Asian, and Pacific Islander]). Socioeconomic covariates included marital status, employment status, education, and total net worth. Socioeconomic covariates were categorized as follows: marital status (never married, married but spouse absent, separated, divorce, widowed; married or living with a partner), current employment status (full time, part-time, partly retired; retired; unemployed, disabled, and not in the labor force), education (less than high school, high school graduate, some college/college graduate, post-college), total net worth (tertile), and household size (1, 2,

© 2025 Lee H et al. *JAMA Network Open.*

≥3 members). Lifestyle and health-related covariates included vigorous activity, smoking, alcohol drinking, energy intake, and body mass index (BMI). These variables were included in the model as follows: energy intake (continuous, kcal/d), vigorous activity (yes or no), smoking (never smoker, ever smoker, current smoker), alcohol consumption (nondrinker, <5g/day, ≥5g/day), and BMI (<25, 25-30, ≥30 kg/m^2). Depressive symptoms at baseline were assessed using the Center for Epidemiologic Studies Depression Scale-8 (CESD-8), and participants with a score ≥5 were classified as having depressive symptoms (yes or no). Participants with physician-diagnosed diabetes, stroke, heart problems, lung disease, or cancer were classified as having a chronic disease (chronic disease history) (yes or no).

5. Statistical analysis

Attrition-adjusted sampling weights were applied to account for the complex survey design of the HRS and the use of analytic subsamples in this study. To account for the exclusion of participants from the 2013 HCNS nutrient total dataset, we generated study-specific adjusted sampling weights. We modeled the probability of inclusion in our analytic sample using logistic regression, with age, sex, race and ethnicity, and education as predictors to estimate propensity scores. To minimize the influence of extreme weights, eligible participants were grouped into deciles based on their estimated propensity scores. The reciprocal of the mean propensity score within each decile was then multiplied by the original HCNS full-sample weight to compute the final adjusted sampling weight used in this study. Weighted Cox proportional hazards regression models were used to examine associations between food insecurity and cognitive impairment risk. The proportional hazards assumption was satisfied based on Schoenfeld residuals. Baseline covariate values were used in all models. Models adjusted for demographic and socioeconomic covariates in multivariable-adjusted model (MV1), adding lifestyle and health-related covariates in MV2, and finally adding chronic disease history and depressive symptoms in MV3. To test effect modification by age, we included interaction terms between food insecurity and age in the fully adjusted model (MV3) and assessed their significance using a likelihood ratio test comparing models with and without the interaction term. Data was analyzed using SAS 9.4; a 2-sided $P < .05$ indicated statistical significance. Analysis was done from November 2024–March 2025.

© 2025 Lee H et al. *JAMA Network Open.*

eReferences.

1. Economic Research Service. USDA. U.S. Household Food Security Survey Module: Six-Item Short Form 2025. https://www.ers.usda.gov/topics/food-nutrition-assistance/food-security-in-the-us/survey-tools. Accessed April 26, 2025.

2. Crimmins, E.M., J.K. Kim, K.M. Langa, and D.R. Weir, Assessment of cognition using surveys and neuropsychological assessment: the Health and Retirement Study and the Aging, Demographics, and Memory Study. *J Gerontol B Psychol Sci Soc Sci*. 2011;66 Suppl 1(Suppl 1):i162-71. doi:10.1093/geronb/gbr048

© 2025 Lee H et al. *JAMA Network Open.*
