# Supplementary material for: Food Insecurity and Risk of Dementia and Cognitive Impairment With No Dementia in US Older Adults
Source: JAMA Netw Open. 2025 Sep 24;8(9):e2533592. doi: 10.1001/jamanetworkopen.2025.33592 (PMC12461400; doi:10.1001/jamanetworkopen.2025.33592)
Supplement: Supplement 2. — Data Sharing Statement [file jamanetwopen-e2533592-s002.pdf]

Data Sharing Statement

Lee. Food Insecurity and Risk of Dementia and Cognitive Impairment With No Dementia in US Older Adults. *JAMA Netw Open*. Published September 24, 2025.
doi:10.1001/jamanetworkopen.2025.33592

Data
Data available: Yes
Data types: Other (please specify)
Additional Information: All data is publicly available through the HRS websites (https://hrsdata.isr.umich.edu/).
How to access data: All data is publicly available through the HRS websites (https://hrsdata.isr.umich.edu/).
When available: With publication

Supporting Documents
Document types: Other (please specify)
Additional Information: Anyone who registers and agrees to the conditions of use through the HRS website (https://hrsdata.isr.umich.edu/)
How to access documents: Anyone who registers and agrees to the conditions of use through the HRS website (https://hrsdata.isr.umich.edu/)
When available: With publication

Additional Information
Who can access the data: Anyone who registers and agrees to the conditions of use through the HRS website (https://hrsdata.isr.umich.edu/)
Types of analyses: For any purpose, Available to registered users through the HRS website without the need for proposal approval.
Mechanisms of data availability: Available to registered users through the HRS website without the need for proposal approval.
Any additional restrictions: N/A
